# Synthesis, Novel Crystal Structure, and *β*-Amyloid Binding Property of Re(I) (tricarbonyl)^+^ EHIDA Analogue

**DOI:** 10.1155/2009/702730

**Published:** 2009-11-08

**Authors:** Yang Yang, Jia Xin Zhang, Lin Zhu, Huabei Zhang

**Affiliations:** Key Laboratory of Radiopharmaceuticals, Beijing Normal University, Ministry of Education, Beijing 100875, China

## Abstract

A neutral compound Re(CO)_3_(L) (L: 2-((2-(2,6-diethylphenylamino)-2-oxoethyl)(2-ethoxy-2-oxoethyl)amino)acetic acid, an IDA analogue) has been synthesized and evaluated for in vitro imaging probes of *β*-amyloid (A*β*) aggregates. Results of X-ray measurement of Re(CO)_3_(L) demonstrated that the coordination mode of Re(CO)_3_(L) was different from that of classical Re/Tc(I) (tricarbonyl)-IDA analogues; the structure of Re(CO)_3_(L) was confirmed by means of infrared spectrum, HPLC-UV, TOF MS, and X-ray measurements (Cambridge Crystallographic Data Centre number is 732731): monoclinic P2_1_/*c*, *a* = 15.6636 (12) Å, *b* = 10.9360 (8) Å, *c* = 27.756 (2) Å, *α* = 90.000 (0)°, *β* = 90.783 (5)°, *γ* = 90.000 (0)°, and *Z* = 8. The binding affinity for *β*-amyloid plaques was assessed by in vitro binding assay using preformed synthetic A*β*_(1–40)_ aggregates. The neutral compound Re(CO)_3_(L) showed binding affinity to A*β* aggregates at micromolar level by fluorescence spectroscopy, and this work will encourage for further exploration of imaging agents labeled by ^99m^Tc(CO)_3_^+^ center as probes for *β*-amyloid plaques in vivo.

## 1. Introduction

Alzheimer's disease (AD) is a neurodegenerative disease characterized by dementia, cognitive impairment, and memory loss, and so far the only definitive confirmation of AD is universally accepted by histopathologic examination of extracellular amyloid plaques comprised of amyloid-beta (A*β*) aggregates and intracellular numerous neurofibrillary tangles in the postmortem brain; furthermore, formation of A*β* aggregates in the brain became the hallmark feature on early pathogenesis of AD before another alternative hypothesis emerged [1]. Several compounds have been selected to be radiolabeled and used in the noninvasive detection of A*β* aggregates by imaging techniques of positron emission tomography (PET) and single photon emission-computed tomography (SPECT); in addition, these representative A*β*-detectors were derivatives of congo red, thioflavin, stilbene, and DDNP (6-dialakylamino-2-naphthylidene), such as ^11^C-PIB, ^125^I-TZDM, ^11^C-SB-13, and ^18^F-FDDNP [2]. However, the progress of SPECT imaging agents labeled by techenetium-99m center fell behind, because of their low rates of penetration through the blood-brain barrier (BBB) [3]. Iminodiacetic acid (IDA) derivatives EHIDA labeled by ^99m^Tc(CO)_3_ has been studied on physicochemical properties and biological evaluation [4, 5], but the structure of technetium(I)-tricarbonyl EHIDA has not been identified. In this paper, we reported the synthesis of Re(CO)_3_(L) (L is esterified EHIDA), and the novel crystal structure of this complex was determined by X-ray measurement: the coordination mode of Re(CO)_3_(L) was different from that of classical Re/Tc(I) (tricarbonyl)-IDA derivatives. The neutral compound Re(CO)_3_(L) showed binding affinity to A*β* aggregates in vitro at micromolar level by fluorescence spectroscopy.

## 2. Experimental

### 2.1. Materials and Preformed Synthetic A*β*(1–40) Aggregates

A*β*_(1~40)_ peptide was purchased from Shang Hai “Supermed” Trade Limited Company. Re(CO)_5_Br, Thioflavin T, Phosphate Buffer Solution (PBS, pH = 7.4), and EDTA were purchased from Sigma-Aldrich Chemical Company. Other chemicals were all purchased from Beijing Chemical Reagents Company. EHIDA was synthesized according to the previous method [6].

**Figure 1:**
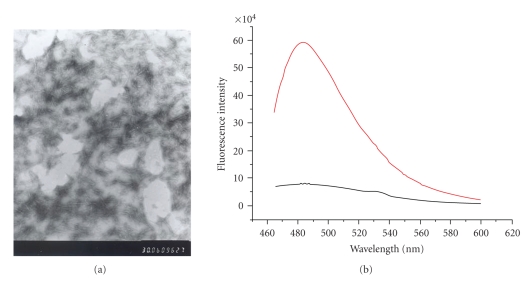
(a) Observation of A*β*_(1~40)_ aggregates by transmission electron microscope (b) Formation of A*β*_(1~40)_ aggregates using Thioflavine T by fluorescence spectroscopy red line: fluorescence intensity of the mixture of 5 *μ*L solution of A*β*_(1~40)_ aggregates and 500 *μ*L solution of Thioflavine T (2 *μ*M); black line: fluorescence intensity of 500 *μ*L solution of Thioflavine T (2 *μ*M) only.

*Formation of A* *β*_(1~40)_ *aggregates*. A*β*_(1~40)_ aggregates (Figure 1(a)) were prepared according to the method published previously [7]. 1 mg of brief A*β*_(1~40)_ and 20 *μ*L EDTA solution (1 mM) were dissolved in 2 mL PBS (pH = 7.4), and the mixture was mixed with a magnetic stir bar (300 rpm) for 72 hours at 37°C to result in a visibly cloudy solution. The production of A*β*_(1~40)_ aggregates was confirmed by a Jeol 100CX-transmission electron microscope (purchased from JEOL USA, Inc.). Additional test for the formation of A*β*_(1~40)_ aggregates was also performed using Thioflavine T (2 *μ*M) by fluorescence spectroscopy (excitation wavelength: 450 nm, scan range was from 460 to 600 nm) [8]; there was an obvious enhance of fluorescence intensity of Thioflavine T at the emission wavelength of 485 nm when 5 *μ*L solution of A*β*_(1~40)_ aggregates was added to the 500 *μ*L solution of Thioflavine T (2 *μ*M) (Figure 1(b)). A*β*_(1~40)_ aggregates were used immediately after preparation.

### 2.2. Syntheses and X-Ray Measurement

EHIDA (2,2′-(2-(2,6-diethylphenylamino)-2-oxoethylazanediyl)diacetic acid) was synthesized according to the previous method [6], and the structure of EHIDA has been confirmed by using melting point mensuration, IR, ^1^H-NMR, ^13^C-NMR, and Mass Spectrometry (data did not be shown). 200 *μ*mol Re(CO)_5_Br reacted with the equimolar EHIDA in the refluxing mixture of 50 mL ethanol and 0.1 mL H_2_SO_4_ (4 mol/L) for 48 hours, then the reaction solution was washed by saturated NaHCO_3_ solution and the target product was extracted by ethyl acetate, and after evaporating the ethyl acetate, the yellowish solid precipitated. The product was recrystallized in the mixture of dichloromethane/ethanol (4 : 1) for one week to obtain Re(CO)_3_(L) crystal. Results of X-ray measurement of Re(CO)_3_(L) were obtained (Tables 1 and 2). IR (KBr): high bands of CO (2036.6, 1922.3, 1903.0 cmv^−1^, very strong) and additional bands for the CO-group of the amide (1633.0 cm^−1^, strong), CH_3_ group (3062.9, 2969.3 cm^−1^, medium). TOF MS^+^ (*m/z*): 619.0880, 621.0872; calcd for C_21_H_25_N_2_O_8_^185/187^Re, M = 619.0, 621.0.

The X-ray measurement of Re(CO)_3_(L) was carried out on a CCD area X-ray detector. The Mo K*α* radiation (wavelength = 0.71073 Å) was used. The data were collected by SADABS (Siemens Area Detector Absorption correction program). The nonhydrogen atoms were refined anisotropically, whereas the hydrogen atoms were placed in the calculated positions. The atomic scattering factors were taken from [9, Tables 6.1.1.4 and 4.2.6.8]. Details of the X-ray measurements and crystal data for Re(CO)_3_(L) are given in Tables 1 and 2. Copies of the data can be obtained on application to CCDC, 12 Union Road, Cambridge CB2 1EZ, UK (Fax: (+44) 1223-336-033; E-mail: deposit@ccdc.cam.ac.uk; Internet: http://www.ccdc.cam.ac.uk/conts/retrieving.html). The CCDC number of Re(CO)_3_(L) is 732731.

### 2.3. HPLC-UV Analysis and UV Visible of Re(CO)_3_(L) and EHIDA

**Figure 2:**
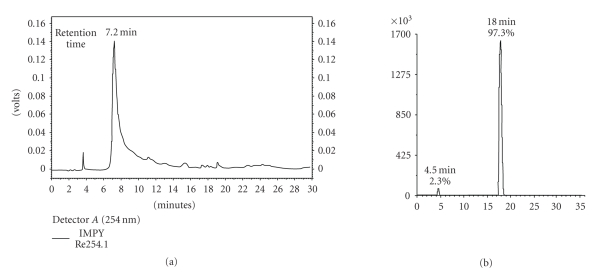
HPLC retention times of Re(CO)_3_(L) (a) and ^99m^Tc(CO)_3_(EHIDA) (b).

**Figure 3:**
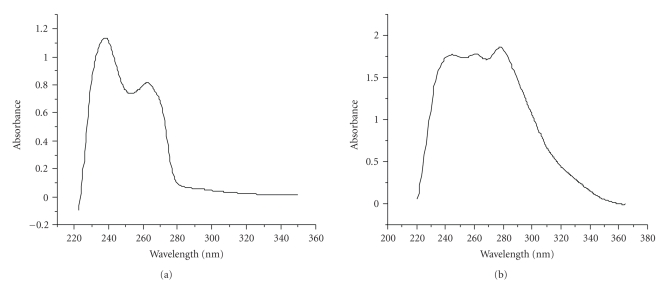
Ultraviolet Spectrum of the EHIDA (a) and Re(CO)_3_(L) (b).

HPLC-UV analysis was carried out with an Alltima RP C-18 column (250 × 4.6 mm^2^, 5 *μ*m) by using an Shimadzu System with SCL-10Avp HPLC pump system and UV detector. The analysis gradient was (a): 0.1% trifluoroacetic acid in water, (b): 0.1% trifluoroacetic acid in acetonitrile: 0 to 20 minutes, from 100% (a) to 100% (b); 20 to 30 minutes, 100% (b). The flow rate was 0.9 mL/min. The excitation wavelength was 254 nm. Results were displayed in Figure 2. UV-visible absorption spectra of the ethanol solution of Re(CO)_3_(L) and EHIDA were recorded, with excitation wavelength scanning from 220 to 360 nm at room temperature. Results were in Figure 3.

### 2.4. Binding Affinity to A*β*_(1~40)_ Aggregates by Fluorescence Spectroscopy

Fresh solution of 0.1 mM Re(CO)_3_(L) was diluted to obtain a final concentration range of 19 ~ 24 *μ*M Re(CO)_3_(L) in 500 *μ*L PBS (pH 7.4). 5 *μ*L solution of A*β*_(1~40)_ aggregates was added to 500 *μ*L PBS solution of Re(CO)_3_(L) (the concentrations were 19, 20, 21, 22, 23, 24 *μ*M, resp.), and the mixed solution was incubated for 1 minute at room temperature before measuring the fluorescence intensity (slit-width was 2 nm). Excitation wavelength was 320 nm, and a 350 ~ 600 nm scan range was performed. All the data were performed in triplicate. The linear relationship between the concentrations (Re(CO)_3_(L) only and Re(CO)_3_(L) incubated with A*β*_(1~40)_ aggregates) and the integration of corresponding fluorescence intensity was analysed by Microcal Origin 6.0. Results were shown in Figure 6.

## 3. Results and Discussion

### 3.1. Comparison of HPLC Analysis between Re(CO)_3_(L) and ^99**m**^Tc(CO)_3_(EHIDA)

The retention time of Re(CO)_3_(L) (7.2 minutes) was not similar to that of ^99m^Tc(CO)_3_(EHIDA) (18.0 minutes) [5] (shown in Figure 2), which is speculated due to the change of lipophilicity of the ligand from EHIDA to esterification form. Esterified EHIDA (L) was more lipophilic than EHIDA; so the retention times of Re(CO)_3_(L) and ^99m^Tc(CO)_3_(EHIDA) were different; however, the difference of properties of the Re(CO)_3_^+^ core and ^99m^Tc(CO)_3_^+^ core may also lead to the different retention times.

### 3.2. UV Visible of Re(CO)_3_(L) and EHIDA

The UV-visible absorption spectra of the complexes are illustrated in Figure 3. Upon EHIDA, the visible intraligand (*π*-π*) bands at 240 nm and 265 nm were due to the fact that these two bands are of mainly bpy-localized in nature. In comparison, the additional visible MLCT (d-π*) band of Re(CO)_3_(L) at 280 nm was clearly associated with the coordinational –C=O group on comparison with the UV spectra of the analogue Re(I)(tricarbonyl)^+^ complexes [10] without the –CH_2_–C=O group.

### 3.3. Description of the Structure of Re(CO)_3_(L): The neutral crystal of Re(CO)_3_

**Figure 4:**
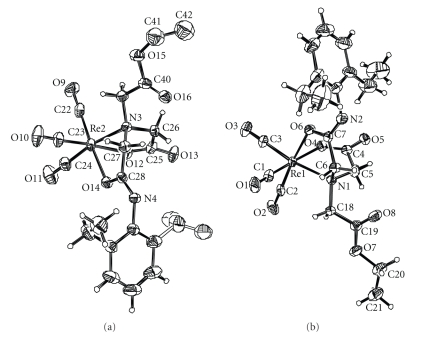
ORTEP drawing of the Re(CO)_3_(L).

Selected distances and bond angles of the neutral crystal of Re(CO)_3_(L) are listed in Table 2, and the molecular views are presented in Figure 4. From the ORTEP drawing of the Re(CO)_3_(L), it can be known that there were two different structures of Re(CO)_3_(L) in one crystal cell.

**Figure 5:**
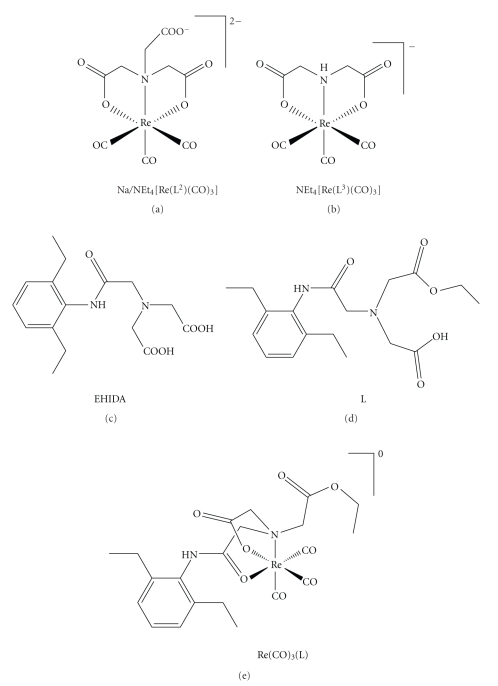
Structures of Na/NEt_4_[Re(L^2^)(CO)_3_], NEt_4_[Re(L^3^)(CO)_3_] [11] EHIDA, L, and Re(CO)_3_(L).

The IDA derivatives often coordinate with Re/Tc(I) (tricarbonyl)^+ ^ centers via classic coordination manner, which is that each oxygen atom of the two carboxyl groups and one N atom combine to the empty orbit in the rhenium or technetium [11], oppositely, our research of the crystal structure of Re(CO)_3_(L) demonstrated that oxygen atom of carbonyl has more stronger coordination ability than the carboxyl group, so that an oxygen atom in the carbonyl group, an oxygen atom in the carboxyl group, and the N atom coordinated with the Re(CO)_3_^+^ core in the molecular of Re(CO)_3_(L), and as a result, 2-((2-(2,6-diethylphenylamino)-2-oxoethyl)(2-ethoxy-2-oxoethyl)amino)acetic acid (L or Esterified EHIDA) reacted with Re(CO)_5_Br resulting in neutral but not negative Re(CO)_3_(L) (Figure 5).

### 3.4. Studies on Binding A*β*_(1~40)_ Aggregates In Vitro

**Figure 6:**
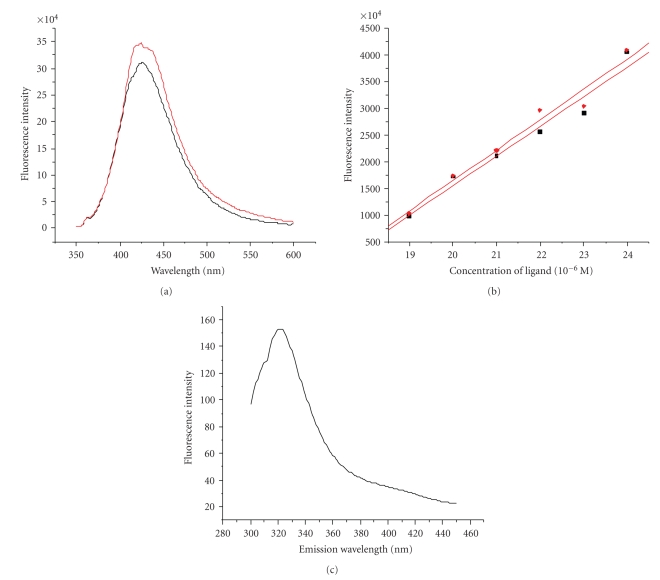
(a) The fluorescence intensity of Re(CO)_3_(L) (20 *μ*M) (black line) and Re(CO)_3_(L) (20 *μ*M) incubated with A*β*_(1~40)_ aggregates (red line) at the emission wavelength of 425 nm. (b) Equation (1): the nether line and Equation (2): the upper line. (c) The fluorescence intensity of EHIDA (0.4 mM) and emission wavelength (excitation wavelength is 280 nm).

The fluorescence intensity of the mixture of A*β*_(1~40) _ aggregates and Re(CO)_3_(L) can be significantly stronger than the fluorescence intensity of Re(CO)_3_(L) only when the concentration of Re(CO)_3_(L) was more than 19 *μ*M; for example, when 500 *μ*L Re(CO)_3_(L) (20 *μ*M) was added to the solution of A*β*_(1~40)_ aggregates, there was an obvious enhance of fluorescence intensity at the emission wavelength of 425 nm (Figure 6(a)), oppositely, different from the visible fluorescence spectroscopy of EHIDA at 320 nm (Figure 6(c)). Upon excitation there occurs an intramolecular charge transfer of Re(CO)_3_(L) between the –NH–C=O group and rhenium, and then back charge transfer will be impossible. Upon addition of A*β*_(1~40)_ aggregates, there was a visible enhance of fluorescence intensity at 425 nm, with no disturb of emission wavelength. Considering the breakage of the system of conjugated bonds due to the reorientation of the benzene ring and amide group of Re(CO)_3_(L) with respect to one another in PBS solution, the enhance of fluorescence intensity was mainly because of the breakage of the system of conjugated bonds being inhabited by the microsurrounding of *β*-amyloid aggregates when A*β*_(1~40)_ aggregates was added [12]; as a result, an increased MLCT of Re(CO)_3_(L) then occurred. However, the dissociation constant of Thioflavin T equaled 2 *μ*M [8], lower than that of Re(CO)_3_(L), because of there is no obvious enhance of fluorescence intensity when the concentration of Re(CO)_3_(L) was little than 19 *μ*M. Generally, probes which bind characteristically to *β*-amyloid fribils have more than one conjugationg system and electron-donating groups such as Me_2_N–, MeNH–, MeO–, and HO– in their molecular skeletons [13], opposite to the structure of Re(CO)_3_(L), indicative of a nonclassical intercalation of the complex binding to the A*β*_(1~40)_ aggregates. The rather good linear relationship between the concentrations of Re(CO)_3_(L) and fluorescence intensity (equation (1), Figure 6(b)) was obtained by Microcal Origin 6.0:

(1) $$\begin{array}{ll} \text{Y} & =-9.51095\times 10^{7}+5.53571\times 10^{6}\text{X}\\ & \;\;\;\;(R\text{:}\;0.98151,\text{SD:}\;2.2583\times 10^{6},\\ & \;\;\;\;N\text{:}\;6,P\text{:}\;5.09878\times 10^{-4}), \end{array}$$

where *Y* is the fluorescence intensity; *X* is the concentrations, from 19 to 24 *μ*M; *R* is the linear correlation coefficient; SD is the standard error.

When binding to *β*-amyloid aggregates, there was also a great linear relationship between the concentrations of Re(CO)_3_(L) and fluorescence intensity (equation (2), Figure 6(b)):

(2) $$\begin{array}{ll} Y & =-9.74219\times 10^{7}+5.69714\times 10^{6}X\\ & \;\;(R\text{:}\;0.98544,\text{SD:}\;2.05598\times 10^{6},\\ & \;N\text{:}\;6,P\text{:}\;3.16429\times 10^{-4}), \end{array}$$

where *Y* is the fluorescence intensity; *X* is the concentrations of Re(CO)_3_(L), from 19 to 24 *μ*M; *R* is the linear correlation coefficient; SD is the standard error.

## 4. Conclusion

In conclusion, the synthesis and X-ray measurement of Re(CO)_3_(L) was successfully completed, providing a novel crystal structure which was not similar to that of classical Re/Tc(I)(tricarbonyl)^+^ IDA derivatives. Evaluation of its binding affinity to A*β*_(1~40)_ aggregates by the fluorescence method demonstrated that the binding characteristic was at micromolar level, which suggested that the structural modification should be achieved for future exploration of ^99m^Tc(CO)_3_^+^ core labeled EHIDA derivatives as imaging agents for A*β*_(1~40)_ plaques in vivo, and this work will encourage for further exploration of probes for *β*-amyloid plaques.

## Figures and Tables

**Table 1:** Summary of crystal data of Re(CO)_3_(L).

	Re(CO)_3_(L)
Empirical formula	C_21_H_19_N_2_O_8_Re
Formula weight	613.58
Temperature	296 (2) K
Wavelength	0.71073 A
Crystal system, space group	Monoclinic, *P*2(1)/c
*Unit cell dimensions*	
*A *(Å)	15.6636 (12)
*B *(Å)	10.9360 (8)
*C *(Å)	27.756 (2)
*α* (°)	90.000 (0)
*β* (°)	90.783 (5)
*γ* (°)	90.000 (0)
*Unit cell Volume *(Å^3^)	4754.1 (6)
*Z*, Calculated density	8, 1.715 mg/m^3^
Absorption coefficient (mm^−1^)	5.158 mm^−1^
*F*(000)	2384
*Crystal size*	0.34 × 0.07 × 0.05 mm
*Completeness to theta = 25.46*	99.5%
Goodness-of-fit on *F* ^2^	1.007
Final *R* indices [*I* > 2*σ*(*I*)]; *R*_1_, *w* *R*_2_	0.0324, 0.0729
*R* indices (all data); *R*_1_, *w* *R*_2_^a^	0.0619, 0.0838

^a^ *R*_1_ = ∑(|*F*_*o*_| − |*F*_*c*_|)/∑|*F*_*o*_|; *w* *R*_2_ = [∑[*w*(*F*_*o*_^2^ − *F*_*c*_^2^)^2^]/∑[*w*(*F*_*o*_^2^)^2^]]^1/2^.

**Table 2:** Selected bond distances (Å) and bond angles (°) for the neutral complex: Re(CO)_3_(L).

Ligand	Re(CO)_3_(L)
Re_1_-C_CO_	1.896 (7), 1.871 (6), 1.912 (6)
Re_2_-C_CO_	1.891 (7), 1.904 (7), 1.918 (7)
Re_1_-O_carboxyl_	2.115 (4), 2.160 (4)
Re_2_-O_carboxyl_	2.109 (4), 2.174 (4)
Re_1_-N	2.254 (4)
Re_2_-N	2.266 (4)
C(2)-Re(1)-C(1)	87.9 (3)
C(2)-Re(1)-C(3)	87.0 (3)
C(1)-Re(1)-C(3)	90.0 (3)
C(2)-Re(1)-O(4)	97.8 (2)
C(1)-Re(1)-O(4)	172.1 (2)
C(3)-Re(1)-O(4)	95.7 (2)
C(2)-Re(1)-O(6)	175.6 (2)
C(1)-Re(1)-O(6)	93.2 (2)
C(3)-Re(1)-O(6)	97.2 (2)
O(4)-Re(1)-O(6)	80.68 (15)
C(2)-Re(1)-N(1)	100.4 (2)
C(1)-Re(1)-N(1)	95.6 (2)
C(3)-Re(1)-N(1)	170.8 (2)
O(4)-Re(1)-N(1)	78.01 (15)
O(6)-Re(1)-N(1)	75.23 (14)
C(22)-Re(2)-C(23)	86.2 (3)
C(22)-Re(2)-C(24)	88.1 (3)
C(23)-Re(2)-C(24)	86.5 (3)
C(22)-Re(2)-O(12)	98.9 (2)
C(23)-Re(2)-O(12)	174.3 (2)
C(24)-Re(2)-O(12)	96.2 (2)
C(22)-Re(2)-O(14)	174.3 (2)
C(23)-Re(2)-O(14)	94.1 (2)
C(24)-Re(2)-O(14)	97.7 (2)
O(12)-Re(2)-O(14)	80.57 (15)
C(22)-Re(2)-N(3)	99.1 (2)
C(23)-Re(2)-N(3)	99.1 (2)
C(24)-Re(2)-N(3)	171.2 (2)
O(12)-Re(2)-N(3)	77.70 (15)
O(14)-Re(2)-N(3)	75.20 (15)
